# Supplementary material for: Interplay between Partial EMT and Cisplatin Resistance as the Drivers for Recurrence in HNSCC
Source: Biomedicines. 2022 Oct 5;10(10):2482. doi: 10.3390/biomedicines10102482 (PMC9598677; doi:10.3390/biomedicines10102482)
Supplement: Supplementary file 1 [file biomedicines-10-02482-s001.zip › biomedicines-1846295-supplementary.pdf]

Supplementary Materials:

Table S1 A. Study population of Slug and KLF4 immunohistochemistry evaluation. N=72.

**gender**	male	63
	female	9
**age**	<=50	3
	51-60	11
	61-70	25
	71-80	22
	>80	11
**tumor type**	Primary	58
	Recurrence	7
	Secondary	7
**Site**	Oral	11
	Larynx	12
	Oropharynx	36
	Hypopharynx	10
	Other	3
**histology**	squamous cell carcinoma	72
**HPV status**	P16-positive (>=70%) *	18
	P16-negative (<70%)	54
**Survival**	alive	53
	deceased	19
**First treatment modality**	Surgical/ Surgical PORT	34
	Best supportive care	3
	R(C)T	35

*HPV-positivity was also confirmed by HPV-specific PCR.

Table S1 B. Patients data of Ki-67 quantitative StrataQuest analysis in 40 regions of interest (ROIs) collected from 7 patients with paired primary and recurrence tumor samples. The clinical data are listed for ROIs and not for patients. N= 40 (ROIs).

**Gender**	male	40
**Age**	<=50	3
	51-60	8
	61-70	15
	71-80	14
	>80	0
**Tumor type**	Primary	16
	Recurrence	24
**Site**	Oral	3
	Larynx	11
	Oropharynx	23
	Other	3
**histology**	squamous cell carcinoma	40
**HPV status**	P16-positive (>=70%)*	0
	P16-negative (<70%)	40
**Survival**	alive	28
	deceased	12
**Treatment modality**	**Primary tumor**	**Recurrence**
Surgical/ Surgical PORT	9	6
Best supportive care		9
R(C)T	7	9

*HPV-positivity was also confirmed by HPV-specific PCR.

Table S2. Overview of utilized Antibodies for IHC.

Antibody	Isotype	catalogue number	manufacturer	dilution
anti-KLF4	rabbit IgG	ab215036	Abcam, Cambridge, UK	1:2000
anti-Slug	mouse IgG1	564614	BD/Pharmingen, Vienna Austria	1:250
anti-Ki67	mouse IgG1	E059	Linaris, Dossenheim, Germany	prediluted
Universal secondary antibody	mouse/rabbit reactive	760-4205	Roche Ventana, Mannheim, Germany	prediluted

Table S3. Overview of utilized primary and secondary antibodies used for western blot analysis.

Antibody	Isotyp	catalogue number	kDa	manufacturer	dilution
anti-KLF4	Rabbit IgG	ab215036	54	Abcam, Cambridge, UK	1:1000
anti-E-cadherin	Mouse IgG2a	610181	120	BD/Biosciences, Vienna Austria	1:2 500
anti-Slug	Rabbit IgG	9585	30	Cell Signaling, Danvers, MA, USA	1:1000
anti-phospho-p38 MAPK	Rabbit IgG	4511S	43	Cell Signaling, Danvers, MA, USA	1:1000
anti-Vimentin (SP-20)	Rabbit IgG	M3202	53	Spring/Linaris, Dossenheim, Germany	1:100
anti-GAPDH	Mouse IgG1	ab8245	36	Abcam, Cambridge, UK	1:5000
Fluorochrome secondary antibody					
αmouse IgG2a 800 CW	IgG2a	926-32351		LiCor Bioscience, Bad Homburg vor der Höhe, Germany	1:5000
α mouse IgG1 680LT	IgG1	926-68050		LiCor Bioscience, Bad	1:10000

				Homburg vor der Höhe, Germany	
α rabbit IgG 800CW	IgG	926-32213		LiCor Bioscience, Bad Homburg vor der Höhe, Germany	1:5000
α mouse IgG IR800	IgG	AC2135		Azure Biotech, Houston, TX, USA	1:2500
Peroxidase secondary antibody					
α mouse IgG HRP	IgG	31430		Pierce, ThermoFisher Scientific, Darmstadt, Germany	1:10000
α rabbit IgG HRP	IgG	31460		Pierce, ThermoFisher Scientific, Darmstadt, Germany	1:10000

Figure S1. StrataQuest Application to determine tumor cells and disseminating tumor cells in Ki-67 immunohistochemical staining.

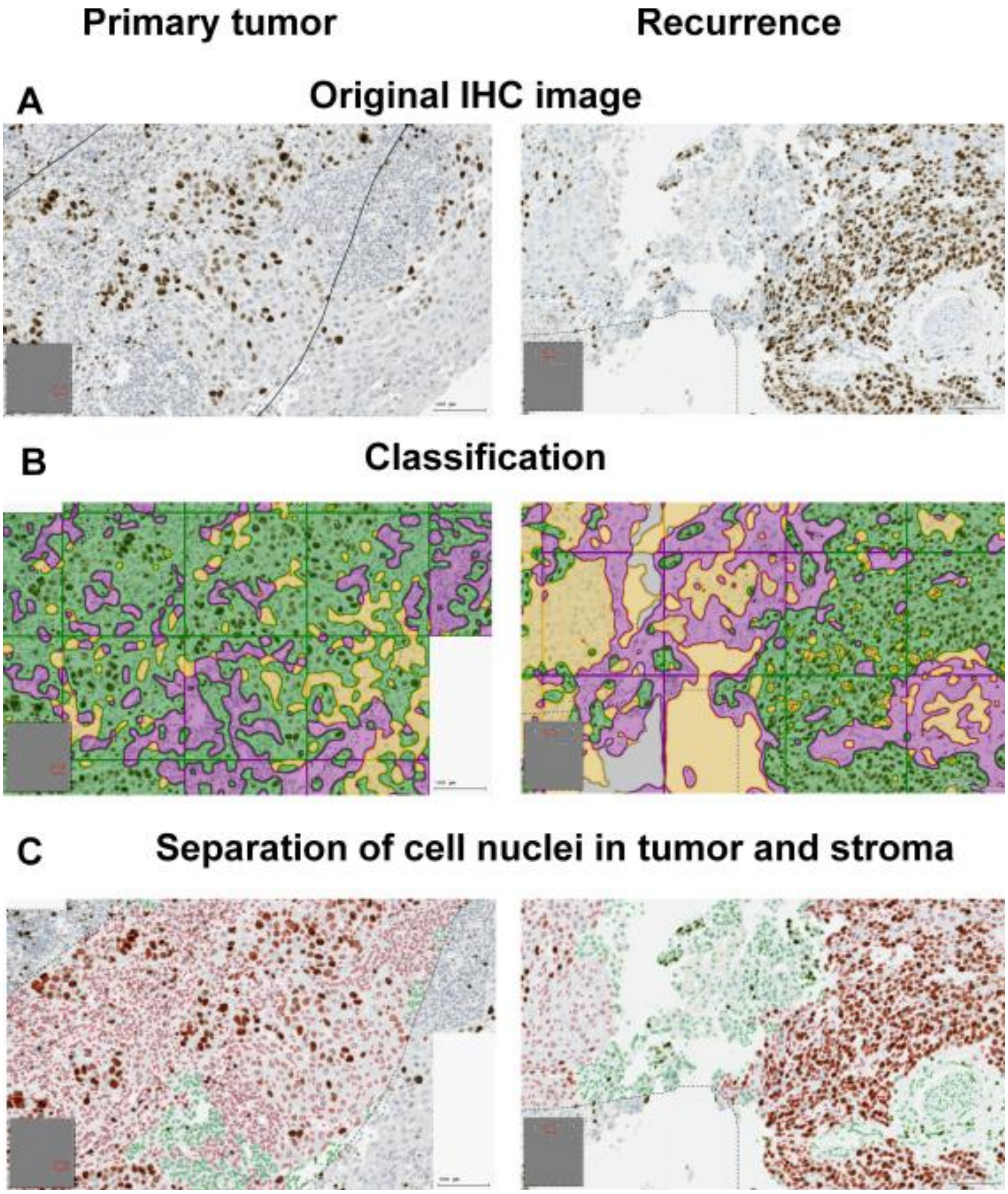

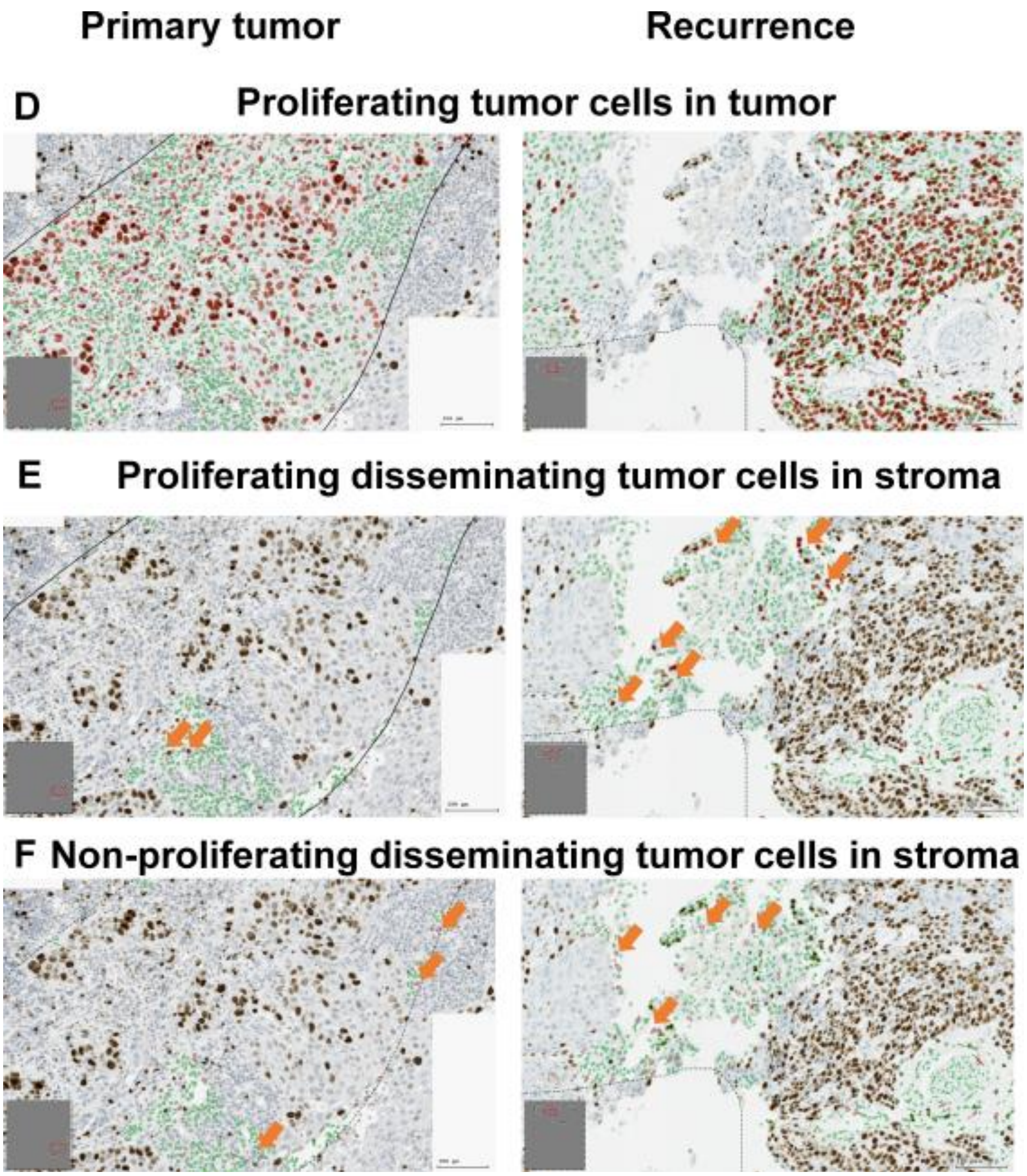

Figure S1. Primary tumor (left panel) and recurrence (right panel) from an oropharynx squamous cell carcinoma. **A**: Original immunohistochemical image **B**: A special developed application of TissueGnostics was purchased and used to determine background (grey), cancer cell nests (green), epithelial (yellow) and stroma (purple) areas by a "Classifier" algorithm. **C**: After classification, the cell nuclei could be sorted to cancer cell nests (red marked nuclei) and to stroma regions (green marked nuclei). In further steps Ki-67-positive (brown DAB-staining in the nuclei) and negative (blue cell nuclei) tumor cell nuclei were gated in cancer cell nests (**D**) and in the stroma (**E**) based on their size, morphology and localization in the classified area. Red labelled shown the Ki-67$^+$ tumor cell nuclei in cancer cell nest (**D**) and in stroma (**E**). Other cells (non-proliferating tumor cells and embedded stroma cells in cancer cell nest in **D**, stroma cells and non-proliferating tumor cells in stroma in **E**) are shown in green. The application could distinguish proliferating and non-proliferating tumor cells and stroma cells in both localisations: cancer cell nests and stroma. **F** shows non-proliferating tumor cells disseminated in stroma in red. In figures **E** and **F** examples of red labelled disseminating tumor cells are also shown with orange arrows. Scale bars represent 100 µm on all

images. On Figure **E** right panel the Ki-67+ clusters of cells embedded in stroma are well visible, which might derive from collective migration of pEMT cells, a point further elucidated experimentally in this article.

Figure S2. Western blot analysis in SCC-25 cells.

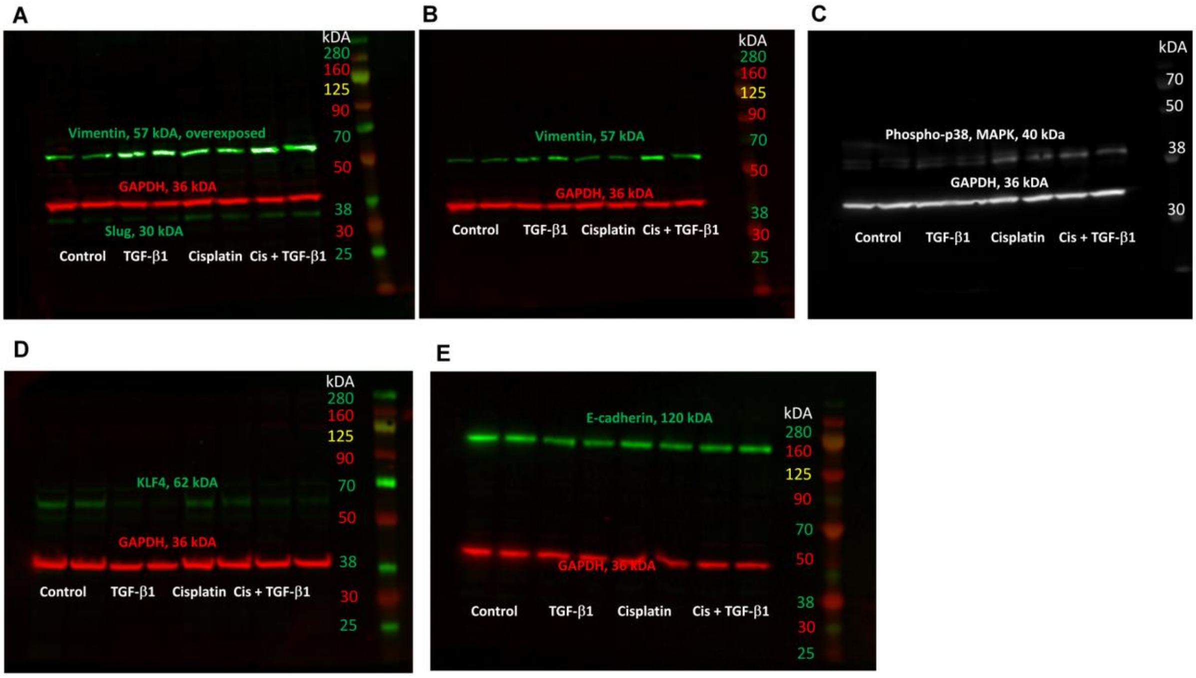

Figure S2. Western Blot of Slug/Vimentin **(A)**, Vimentin **(B)**, phospho-p38 **(C)**, KLF4 **(D)** and E-cadherin **(E)** after TGF-beta1, and Cisplatin treatments. The proteins of interest are presented in green and were detected at 800 nm, the loading control GAPDH is presented in red and was detected at 700 nm **(A, B, D, E)**. Panel **C** is a combination of the chemiluminescence detection of p-p38 and the near infrared detection of GAPDH. Thirty thousand SCC-25 cells per ml were plated on six-well-plates. After 24 hours all cells were supplied with serum-free DMEM-F12 medium (serum proteins were replaced with bovine serum albumin). From day 2 until day 5 cells were treated with serum-free medium optionally supplemented with 1 ng/ml TGF-beta1. From day 5 until day 8 cells were treated with serum-free medium optionally supplemented with 1 ng/ml TGF-beta1, or with 10 µM Cisplatin or with TGF-beta1 and Cisplatin combined as indicated on the images.

Figure S3. Western blot analysis in UPCI-SCC-090 cells.

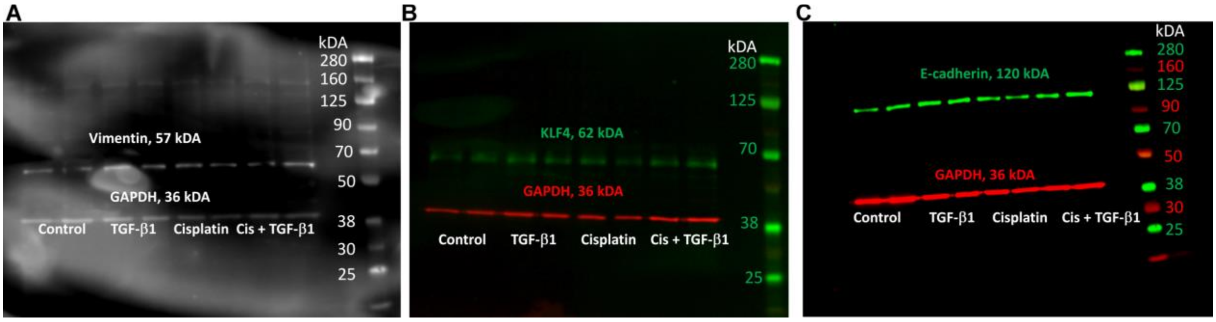

Figure S3. Thirty thousand UPCI-SCC090 cells per ml were plated on six-well-plates. After 24 hours all cells were supplied with serum-free EMEM medium (serum proteins were replaced with bovine serum albumin). From day 2 until day 5 cells were treated with serum-free medium supplemented with 1 ng/ml TGF-beta1 in some cases. From day 5 until day 8 cells were treated with serum-free medium in some cases supplemented with 1 ng/ml TGF-beta1, or with 7 µM Cisplatin or with TGF-beta1 and Cisplatin combined as indicated on the figures. Protein levels of Vimentin **(A)** as EMT factor, KLF4 and E-cadherin **(B, C)** as epithelial markers were analysed by western blotting, GAPDH was used as loading control. All experiments were repeated as complete three biological repeats. Panel **A** was developed by chemiluminescence. The proteins of interest are presented in green and were detected at 800 nm, the loading control GAPDH is presented in red and was detected at 700 nm (**B, C**). In UPCI-SCC090 cells no change in epithelial markers were detected by TGF-beta1 of Cisplatin treatments. Vimentin was very low detected using chemiluminescence and did not show any reaction on TGF-beta1 or Cisplatin treatments. Slug or p-MAPK were not detected at all.

Figure S4. Western blot analysis in SCC-25 cells in complex experiment of TGF-beta-1-pretreatment, KLF4-overexpression, and 9 days after Cisplatin-treatment.

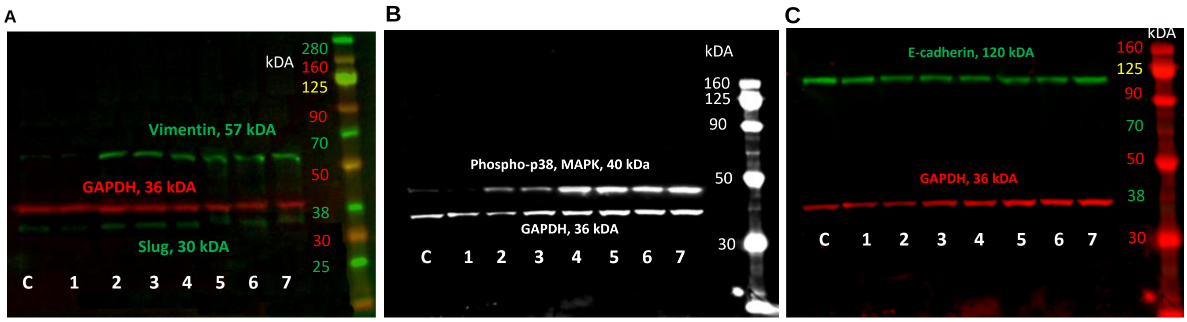

	C	1	2	3	4	5	6	7
**KLF4 overexpression**		+		+		+		+
**1 ng/ml TGF-beta-1-pretreatment**			+	+			+	+
**Cisplatin 10 µM 9 days**					+	+	+	+

Figure S4. Seven thousand SCC-25 cells per ml were plated on six-well-plates. After 24 hours all cells were supplied with serum-free DMEM-F12 medium (serum proteins were replaced with bovine serum albumin). From day 2 until day 6 and from day 6 until day 8 cells were treated with serum-free medium or with 1 ng/ml TGF-beta1 (**lower panel; treatments table**). On days 8 and 9 cells were transfected with expression vectors (empty vector and KLF4-overexpressing vector) and further cultured from day 10 until 13 with 10% serum-containing DMEM-F12 medium. On day 13 cells were 24 hours treated with 10 µM Cisplatin, passaged and replated at 3×10^4 cells/ml (**lower panel; treatments table**). Cells were cultured for 9 days, and used for protein analysis. Typical western blots of Slug/Vimentin **(A)**, phospho-p38 (MAPK) **(B)** and E-cadherin **(C)** three days after transfection in control (serum-free-pretreated), empty vector transfected cells ("C" in images **A-C**) in serum-free-pretreated KLF4-transfected cells (1), in TGF-beta1-pretreated empty vector transfected cells (2), in TGF-beta1-pretreated KLF4 vector transfected cells (3); and 9 days after Cisplatin treatment in control (serum-free-pretreated), empty vector transfected cells (4) in serum-free-pretreated KLF4-transfected cells (5), in TGF-beta1-pretreated empty vector transfected cells (6), in TGF-beta1-pretreated KLF4 vector transfected cells (7). The proteins of interest are presented in green and were detected at 800 nm, the loading control GAPDH is presented in red and was detected at 700 nm **(A, C)**. Panel **B** is a combination of the chemiluminescence detection of p-p38 and the near infrared detection of GAPDH.

The treatments and sample schedules are summarized in **lower panel; treatments table**.

Document S1: On in silico analysis of the SLUG Promoter.

Our in silico analysis using the Ensembl databank (European Molecular Biology Laboratory's European Bioinformatics Institute, on the Wellcome Genome Campus in Hinxton, south of the city of Cambridge, United Kingdom.) revealed a potential of direct regulation of Slug by KLF4.

Slug promoter contains KLF4 responsive elements:

Reference Ensembl site:

http://www.ensembl.org/Homo_sapiens/Transcript/Exons?db=core;g=ENSG00000019549;r=8:48917598-48921740;t=ENST00000020945

The KLF4 binding core sequence is:

GGGTG in antisense orientation in the Slug UTR region.

```
ctttcttgcaaaagagaggaaaaaaaaaccctcccagccaaaacgggctc
AGTTCGTAAAGGAGCCGGGTGACTTCAGAGGCGCCGGCCCGTCCGTCTGCCGCACCTGAG
CACGGCCCCTGCCCGAGCCTGGCCCGCCGCGATGCTGTAGGGACCGCCGTGTCCTCCCGC
CGGACCGTTATCCGCGCCGGGCGCCCGCCAGACCCGCTGGCAAG
```

In addition, KLF4 responsive sequences are also found in sense orientation in the Intron3: TTTTTTTCTCCCACTC**CACCC**CCAG and also in the 3´untranslated region:

```
GTGACGCAATCAATGTTTACTCGAACAGAATGCATTTCTTCACTCCGAAGCCAAATGA
CAAATAAAGTCCAAAGGCATTTTCTCCTGTGCTGACCAACCAAATAATATGTATAGACAC
ACACACATATGCACACACACACACACACACCCACAGAGAGAGAGCTGCAAGAGCATGGAA
TTCATGTGTTTAAAGATAATCCTTTCCATGTGAAGTTTAAAATTACTATATATTTGCTGA
TGGCTAGATTGAGAGAATAAAAGACAGTAACCTTTCTCTTCAAAGATAAAATGAAAAGCA
CATTGCATCTTTTCTTCCTAAAAAAATGCAAAGATTTACATTGCTGCCAAATCATTTCAA
CTGAAAAGAACAGTATTGCTTTGTAATAGAGTCTGTAATAGGATTTCCCATAGGAAGAGA
TCTGCCAGACGCGAACTCAGGTGCCTTAAAAAGTATTCCAAGTTTACTCCATTACATGTC
GGTTGTCTGGTTGCCATTGTTGAACTAAAGCCTTTTTTTGATTACCTGTAGTGCTTTAAA
GTATATTTTTAAAAGGGAGGAAAAAAATAACAAGAACAAAACACAGGAGAATGTATTAAA
AGTATTTTTGTTTTGTTTTGTTTTTGCCAATTAACAGTATGTGCCTTGGGGGAGGAGGGA
AAGATTAGCTTTGAACATTCCTGGCGCATGCTCCATTGTCTTACTATTTTAAAACATTTT
AATAATTTTTGAAAATTAATTAAAGATGGGAATAAGTGCAAAAGAGGATTCTTACAAATT
CATTAATGTACTTAAACTATTTCAAATGCATACCACAAATGCAATAATACAATACCCCTT
CCAAGTGCCTTTTTAAATTGTATAGTTGATGAGTCAATGTAAATTTGTGTTTATTTTTAT
ATGATTGAATGAGTTCTGTATGAAACTGAGATGTTGTCTATAGCTATGTCTATAAACAAC
CTGAAGACTTGTGAAATCAATGTTTCTTTTTTAAAAAACAATTTTCAAGTTTTTTTTACA
ATAAACAGTTTTGATTTAAAATCTCGTTTGTATACTATTTTCAGAGACTTTACTTGCTTC
ATGATTAGTACCAAACCACTGTACAAAGAATTGTTTGTTAACAAGAAAAAAATGAATAAT
GCTTATTATGCATCTGAAGTGTTATTTTATGTGTTAGATTATAATAAAGTAAGCTGCTAG
TATTATTTTTA
```

Graphical presentation of KLF4 binding positions around the Slug gene, chromosomal area.

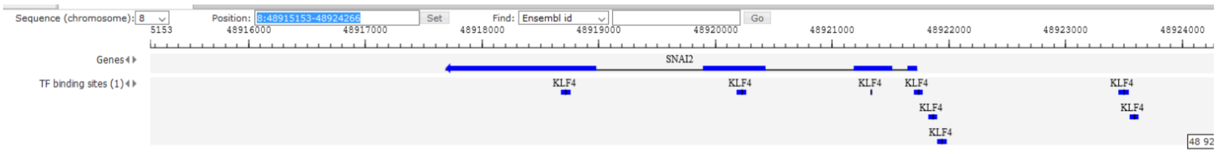

Reference for the KLF4-binding motif: Kaczynski J, Cook T, and Urrutia R (2003) Sp1- and Kruppel-like transcription factors. Genome Biol. 4, 206.
